# Influence of genetic factors on toluene diisocyanate-related symptoms: evidence from a cross-sectional study

**DOI:** 10.1186/1476-069X-7-15

**Published:** 2008-04-30

**Authors:** Karin Broberg, Håkan Tinnerberg, Anna Axmon, Margareta Warholm, Agneta Rannug, Margareta Littorin

**Affiliations:** 1 Department of Occupational and Environmental medicine, Lund University, Sweden; 2 Department of Work Environment Toxicology, Institute of Environmental Medicine, Karolinska Institutet, Sweden

## Abstract

**Background:**

Toluene diisocyanate (TDI) is a highly reactive compound used in the production of, e.g., polyurethane foams and paints. TDI is known to cause respiratory symptoms and diseases. Because TDI causes symptoms in only a fraction of exposed workers, genetic factors may play a key role in disease susceptibility.

**Methods:**

Workers (N = 132) exposed to TDI and a non-exposed group (N = 114) were analyzed for genotype (metabolising genes: CYP1A1*2A, CYP1A1*2B, GSTM1*O, GSTM3*B, *GSTP1 *I105V, *GSTP1 *A114V, GSTT1*O, *MPO *-463, NAT1*3, *4, *10, *11, *14, *15, NAT2*5, *6, *7, *SULT1A1 *R213H; immune-related genes: *CCL5 *-403, HLA-DQB1*05, *TNF *-308, *TNF *-863) and symptoms of the eyes, upper and lower airways (based on structured interviews).

**Results:**

For three polymorphisms: CYP1A1*2A, CYP1A1*2B, and *TNF *-308 there was a pattern consistent with interaction between genotype and TDI exposure status for the majority of symptoms investigated, although it did reach statistical significance only for some symptoms: among TDI-exposed workers, the *CYP1A1 *variant carriers had increased risk (CYP1A1*2A and eye symptoms: variant carriers OR 2.0 95% CI 0.68–6.1, p-value for interaction 0.048; CYP1A1*2B and wheeze: IV carriers OR = 12, 1.4–110, p-value for interaction 0.057). TDI-exposed individuals with *TNF*-308 A were protected against the majority of symptoms, but it did not reach statistical significance. In the non-exposed group, however, *TNF *-308 A carriers showed higher risk of the majority of symptoms (eye symptoms: variant carriers OR = 2.8, 1.1–7.1, p-value for interaction 0.12; dry cough OR = 2.2, 0.69–7.2, p-value for interaction 0.036). Individuals with *SULT1A1 *213H had reduced risk both in the exposed and non-exposed groups. Other polymorphisms, showed associations to certain symptoms: among TDI-exposed,NAT1*10 carriers had a higher risk of eye symptoms and *CCL5 *-403 AG+AA as well as HLA-DQB1 *05 carriers displayed increased risk of symptoms of the lower airways. *GSTM1*, *GSTM3 *and *GSTP1 *only displayed effects on symptoms of the lower airways in the non-exposed group.

**Conclusion:**

Specific gene-TDI interactions for symptoms of the eyes and lower airways appear to exist. The results suggest different mechanisms for TDI- and non-TDI-related symptoms of the eyes and lower airways.

## Background

Exposure to diisocyanates is well known to cause asthma and symptoms such as cough, wheezing, and dyspnoea [1]. Moreover, exposure to diisocyanates also often causes symptoms from the upper airways such as rhinitis and conjunctival irritation [2]. In a recent study we have shown that TDI exposure can affect risk of symptoms from the eyes, nose bleed, and symptoms of the lower airways (odds ratios between 1.8–3.7) [3].

Because diisocyanates cause asthma in only a minor fraction of exposed workers, genetic factors probably modify disease susceptibility [4]. The metabolism of TDI is not yet understood, but there is evidence that several different metabolic pathways are involved in its biotransformation. Isocyanates may react with intracellular glutathione, either directly or through catalysis by the glutathione transferase (GST) system. In the group of GST enzymes that were analyzed, GSTP1 and GSTM1 were found to be the most efficient catalysts of glutathione conjugation of several different isothiocyanates [5]. Moreover, GSTs are involved in the protection against reactive oxygen, which is a key component of inflammation.

Non-enzymatic hydrolysis converts isocyanates to their corresponding amines. There is ample proof that aromatic and heterocyclic amines are activated by oxidation of the primary amine by cytochrome P450 enzymes to aryl hydroxylamines. The most important oxygenase is CYP1A2, but also CYP1A1 and CYP1B1 can activate a range of arylamines to reactive intermediates [6]. Another frequent step in arylamine metabolism is the generation of acetoxy esters by N-acetyltransferases (NAT1 and NAT2). NAT1 is more abundant in the lung as well as in the eye, but low levels of NAT2 have also been reported in these tissues [7,8]. An additional step in the biotransformation of many aromatic amines is sulfatation by cytosolic sulfotransferases (SULTs). A peroxidase-catalysed mechanism, possibly through myeloperoxidase (MPO) in neutrophiles, to activate arylamines has been suggested [9]. Proof for neutrophilic activation in occupational asthma has been reported [10]. Functional polymorphisms in such metabolic genes affecting the metabolism of diisocyanates have been studied for their impact on isocyanate-related symptoms [11-14].

There are also several immune-related genes of relevance for the susceptibility to respiratory diseases. The chemokine CCL5 (also labeled RANTES) is responsible for the recruitment of inflammatory cells, such as eosinophils and T-lymphocytes. The HLA class II molecules are involved in the immune responses as they bind antigen-derived peptides and present them to lymphocytes via the T-cell receptor. Mapp et al. have shown overrepresentation of the DQB1*0503 allele among subjects with TDI-induced asthma [15]. Tumor necrosis factor alfa (TNF) is chronically expressed in TDI-induced asthma [16] and a study in TNF receptor knockout mice has shown that these animals fail to develop immunological effects from TDI [17].

The objective of the present study was to investigate whether the associations of TDI and symptoms of the eyes and airways were modified by relevant functional polymorphisms in metabolizing enzymes and/or in proteins involved in airway inflammation. Thus, a group of workers exposed to isocyanates, mainly TDI, and a non-exposed group, not exposed to any isocyanates, were analyzed for genotype and health effects.

## Materials and methods

### Plants and exposure

The study encompassed eleven plants in the southern part of Sweden. These plants are described in detail in Sennbro et al. [18]. Plants selected were those 13 which were expected to have the highest TDI exposure according to an earlier investigation (referred to in [18]) and where at least three employees worked with polyurethane and/or isocyanates; 11 plants where TDI or TDI-based polyurethane was used in the manufacturing process agreed to participate: five moulding plants, two continuous foaming plants, two flame lamination plants, and two plants with low- or non-heating processes. In four of the five moulding plants, MDI was also used in the manufacturing process although the exposures in air were low or non-detectable. Additionally, in one of these four plants isophorone diisocyanate was utilized.

### Subjects

There were in total 184 occupationally TDI-exposed workers employed at the plants and 132 workers participated in the study. Out of a total 198 employees from five different facilities in southern Sweden, a group consisting of 114 workers, not occupationally exposed to isocyanates or performing any plastic or rubber work, was included as well. The pre-requisites to be included in the study were that participants had agreed to take part in the genetic analysis and that they were present at work/at the work shift when the medical examinations took place. The main limiting factor for inclusion in the study has been absence at work shift on the day that the medical examination took place.

The characteristics of exposed and non-exposed workers are presented in Table 1.

**Table 1:** Characteristics of the study subjects^a^.

		Exposed workers		Non-exposed workers	
		
		N	%	N	%
Sex^b**^	Male/Female	107/25	81/19	55/59	48/52
Smoker	No+Former/Yes	77/52	60/40	71/42	63/37
Atopy	Negative/Positive	94/37	72/28	82/31	73/27
					
*SYMPTOMS*					
Eye symptoms^b**^	No/Yes	71/57	55/45	89/25	78/22
Wheeze etc.	No/Yes	88/41	68/32	90/24	79/21
Dry cough^b**^	No/Yes	88/39	69/31	101/13	89/11
Cough with mucus	No/Yes	84/44	66/34	87/27	76/24
Nose bleed^b**^	No/Yes	96/28	78/23	107/7	94/6
					
*GENOTYPES/PHENOTYPES*					
*GSTM1*	+/-	65/67	49/51	65/49	57/43
*GSTT1*	+/-	110/22	83/17	97/17	85/15
*GSTM3*	AA/AB/BB	94/36/2	71/27/2	78/31/5	68/27/4
GSTP1*105	II/IV/VV	68/51/13	52/39/10	57/46/11	50/40/10
GSTP1*114	AA/AV	114/18	86/14	103/11	90/10
GSTP1*105 +114	A/B/C	68/46/18	51/35/14	57/46/11	50/40/10
*NAT1 *phenotype	S/IM/R	3/88/41	2/67/31	3/76/35	3/67/30
*NAT2 *phenotype	S/IM/R	89/33/10	67/25/8	73/38/3	64/33/3
*MPO*^b**^	GG/GA/AA	97/29/6	73/22/5	74/40/0	65/35/0
*SULT1A1*	GG/GA/AA	43/77/12	33/58/9	46/54/14	40/47/12
CYP1A1*2A	A/B/C	116/14/2	88/11/2	97/16/1	85/14/1
CYP1A1*2B	AA/AG/GG	126/5/1	95/4/1	107/7/0	94/6/0
*TNF*-308	GG/GA/AA	83/35/4	68/29/3	69/35/5	63/32/5
*TNF*-863	CC/CA/AA	93/28/6	73/22/5	78/32/2	70/29/2
*CCL5*	GG/GA/AA	84/38/5	66/30/4	72/36/4	64/32/4
HLA-DQB1*05	-/+	88/39	69/31	87/25	78/22
HLA-DQB1*0501^b*^	-/+	98/29	77/23	98/14	88/12
HLA-DQB1*0502	-/+	118/9	93/7	105/7	94/6
HLA-DQB1*0503	-/+	125/2	98/2	108/4	96/4
HLA-DQB1*0504	-/+	126/1	99/1	112/0	100/0

^a^Data was incomplete for some individuals studied, but these study subjects were included in the analyses when possible. Due to variations in success rate of the genotype analyses, the number of analyzed individuals differs to some extent between the different polymorphisms. Moreover, all 246 individuals approved analysis of metabolizing genes, whereas 239 individuals approved analysis of immune-related genes.

^b^Statistically significant differences between exposed and non-exposed workers.

*p < 0.05, **p < 0.01.

The median age in the exposed group was 36 years (range 18–64), and in the non-exposed group 39 (18–61). The non-exposed group has been described in more detail in Sennbro et al. [19]; they did not handle any isocyanates, polyurethanes or other plastics. Moreover, they were not involved in heating operations. This study was approved by the Regional Ethics Committee at Lund University, Sweden. Written informed consent was obtained from all subjects.

### Health examinations

The health examinations are described in detail in Littorin et al. [3]. In the present study we have only focused on symptoms which were shown by Littorin et al. [3] to be significantly associated with TDI exposure. In short, a physician compiled thorough medical and occupational histories from questionnaires with interviews. Symptoms during the last twelve months were recorded and i.a. the following symptoms were registered: symptoms from the eyes, symptoms from lower airways and nose bleed (Table 1). At least one of itching, running, or burning was defined as symptoms of the eyes. Cough with mucus, attacks of severe dry cough, attacks of at least one of dyspnoea, wheezing, or chest tightness were defined as symptoms of the "lower airways" (dyspnoea, wheezing, or chest tightness are in the following text labeled as "wheeze etc."). The employees were physically examined during workdays. Venous blood and urinary samples were collected at work and stored at -20°C. Blood was analyzed for genotypes, plasma and urine for biomarkers of isocyanates. Serum was screened for atopy by the Phadiatop fluoroimmunoassay test [20], which was considered positive when at least one of 11 airborne allergens [dog, cat, horse, timothy, birch, mugwort, mite (D. pteronyssinus, D. farinae), mould (Cladosporium), olive, Parietaria] provided a response. The test has a sensitivity and a specificity of 95% (according to the company supplying the test).

### Exposure assessment methods

Personal exposure was monitored by air measurements and by analysis of biomarkers of exposure in urine and plasma [21,22]. The exposed subjects were exposed to measurable concentrations of airborne TDI (the personal 8-hour time-weighted average levels were for 2,4 TDI: median 0.32 ppb, range below limit of detection (LOD)-2.6 ppb; 2,6-TDI: median 0.27 ppb [0.01–3.6]). The exposed workers had detectable levels of biomarkers in urine (2,4-TDA: median 23 nmol/L [LOD-620]; 2,6-TDA: median 30 nmol/L [LOD-350 nmol/L]) and in plasma (2,4-TDA: median 38 nmol/L [LOD-250]; 2,6-TDA: median 35 nmol/L [LOD-510]); for both analyses LOD = 0.41 nmol/L. No air monitoring was performed in the group of non-exposed workers. However, as much as 15% of them had detectable, but very low, concentrations of biomarkers in urine (2,4-TDA median LOD nmol/L [LOD-3.3]; 2,6-TDA median LOD nmol/L [LOD-1.6]) or plasma (2,4-TDA median LOD nmol/L [LOD -0.82]; 2,6-TDA median LOD nmol/L [LOD-0.82]) respectively, reflecting background exposure [19].

### Genotyping

DNA for genotyping was prepared from white blood cells. Due to variations in success rate of the genotype analyses, the number of analyzed individuals differs to some extent between the different polymorphisms. Moreover, all 246 individuals approved analysis of metabolizing genes, whereas 239 individuals approved analysis of immune-related genes.

Two closely linked single nucleotide polymorphisms (SNPs) in the *CYP1A1 *gene were analyzed by PCR, as previously described [23]. The *Msp*I restriction fragment length polymorphism in the 3'end of *CYP1A1 *(found on the CYP1A1*2A allele) is due to a C>T base substitution 264 base pairs downstream the poly(A) signal. The CYP1A1*2B allele (A>G) contains an additional replacement of isoleucine by valine at residue 462 in the heme binding region of the enzyme.

Genotyping for the *GSTM1 *and *GSTT1 *null, and *GSTP1 *I105V (A>G) polymorphisms was performed as previously described [23]. The *GSTP1 *114 Ala (A) to Val (V) substitution was analyzed by PCR and Dynamic Allele Specific Hybridization (DASH) [24], using 5'-biotin-AGTAGGATGATACATGGTGGTG as the forward and 5'-GCAGTGCCTTCACATAGTCATC as the reverse PCR primer, and 5'-CCTTGCCCGCCTCCTGCC and 5'-CCTTGCCCACCTCCTGCC as specific probes for the C (Ala) and T (Val) alleles, respectively. When combining the genotypes at both positions three groups were formed; A: II+AA at position 105 and 114, respectively; B: IV+AA or VV+AA; and C: IV+AV or VV+AV. The 3 bp deletion (intron 6) of *GSTM3 *was analyzed by PCR/RFLP using the restriction enzyme *Mnl*I according to Inskip et al [25].

Five different SNPs in the *NAT1 *gene, 559 C>T, 560 G>A, 640 T>G, 1088 T>A and 1095 C>A, were analyzed to determine the alleles *3 (1095 C>A), *4 (wild type), *10 (1088 T>A and 1095 C>A), *11 (640 T>G), *14 (560 G>A) and *15 (559 C>T), essentially as described by Deitz et al [26]. However, the 560 SNP was analyzed by allele-specific PCR using 5'-GTTAATTTCTGGGAAGGATCA as the forward primer, and 5'-GAGTAAAGGAGTAGATTTTC and 5'-GAGTAAAGGAGTAGATTTTT, as reverse primers, to differentiate the G and A alleles. Individuals homozygous or heterozygous for the NAT1*10 allele and not carrying the *14 or *15 alleles were considered to have the rapid acetylator phenotype (R). Individuals homozygous for NAT1*3, *4 or *11 alleles were considered to have the intermediate phenotype (IM). The slow acetylator alleles NAT1*14 and *15 were rare in the study population: there were two individuals carrying *10*14 and *10*15 and four individuals carrying *4*14 and all were considered as slow acetylators (S). *NAT2 *polymorphisms (341 T>C, 590 G>A, and 857 G>A) were analyzed as previously described [27]. The *NAT2 *phenotype was deduced from the genotype and subjects were classified as slow (S = carriers of two mutations), intermediate (IM = carriers of one mutation) or rapid (R = carriers of wild type alleles only) acetylators.

For the *MPO *G-463A polymorphism, a PCR/RLFP method with *Aci*I was used [28]. The *SULT1A1*, Arg213His (R213H) was analyzed by PCR/RLFP using the restriction enzyme *Bsp143*II to distinguish the G and A alleles according to Engelke et al [29].

Analysis of *CCL5 *-403G/A was performed with a Taqman-based assay (ABI PRISM 7000 instrument, according to a standard protocol (Applied Biosystems)), forward (5'-CACTTTATAAATAACATCCTTCCATGGAT) and reverse primers (5'-TCAAAGTTCCTGCTTATTCATTACAGA), MGB-probes (5'FAM-AGGGAAAGGAGGTAAGA for the G-allele, and 5'VIC-AGGGAAAGGAGATAAG for the less common A-allele), as well as 20 ng of template. The two polymorphisms in *TNF *(-308 G/A and -863 C/A) were analyzed with matrix-assisted laser desorption/ionization time-of-flight (MALDI-TOF) mass spectrometry (Sequenom™, San Diego, CA, USA) at the SWEGENE Resource Center for Profiling Polygenic Disease, Lund University, Malmö. HLA-DQB1*05-genotype was assessed with sequence-specific primers and amplification control primers as previously reported [30].

In all types of genetic assays, positive controls for each genotype as well as a negative control (no template) were included. Furthermore, for all assays at least 5% of the samples were reanalyzed and the agreement between these analyses was always 100%.

### Statistical methods

The Hardy Weinberg equilibrium, which is the principle for the relation between frequencies of alleles and the genotype of a population was here used for indication of genotyping errors and was tested for all genes with the Chi-square test. The difference in characteristics for the study subjects was evaluated with non-parametric tests (Mann-Whitney or Kruskal Wallis). The genetic influence on the symptoms was evaluated by comparing different genotypes among exposed and non-exposed separately in logistic regression analysis. In a further analysis, interaction between genotype and exposure status (exposed/non-exposed) was evaluated using logistic regression with an interaction term (genotype)*(exposure). Effects were estimated using odd ratios (ORs) together with 95% confidence intervals (CIs). Statistical significance refers to P < 0.05 (two-tailed) or, equivalently, to 95% CI for an OR that excludes 1.0. Unadjusted as well as adjusted analyses were performed. Based on the general knowledge of effects on symptoms of the airways and the eyes, we adjusted for age, sex, smoking (nonsmoker + former smoker vs. smoker), and atopy. Due to missing information, 112 non-exposed and 128 exposed workers could be included in the adjusted analysis compared to 114 non-exposed and 132 exposed workers in the unadjusted analyses. The genotypes of *GSTM1*, *GSTT1*, *NAT1 *and *NAT2 *were dichotomized according to the inferred phenotype: present vs. null for *GSTM1 *and *GSTT1*, slow+intermediate vs rapid for *NAT1 *or intermediate+rapid vs slow, and slow vs. rapid+intermediate acetylation for *NAT2*. The main effects of the remaining genes were first assessed separately for each genotype; genotypes were subsequently grouped based on prior biological knowledge of the allele function or the frequency distribution in our material. For analysis of gene-gene interactions, the analysis was limited to genotypes that were significantly associated with symptoms in the single gene analysis. For example for symptoms of the eyes, the combinations of *NAT1 *(categorized as IM+S/R)+*SULT1A1 *(GG/AG+AA), *NAT1*+*TNF*-308 (GG/AG+AA), and *SULT1A1*+*TNF*-308 were evaluated. All gene-gene combination analyses were adjusted for sex, age, atopy and smoking.

The data were analysed for patterns of associations between genetic markers and different symptoms and such patterns were presented, although some effect estimates/interaction terms did not reach statistical significance. For genetic markers with no obvious pattern of associations, only those resulting in significant effect estimates/interaction terms were presented.

## Results

There were statistically significant differences for sex (p-value < 0.001), genotype distributions for *MPO *(p = 0.008) and HLADQB1*0501 (p = 0.038) between exposed and non-exposed groups (Table 1). Also there were statistically significant or near significant differences in prevalence of symptoms between the two groups (symptoms of the eyes p < 0.001; wheezing p = 0.060; dry cough p < 0.001; cough with mucus p = 0.069; and nose bleed p < 0.001), which has been reported elsewhere [3]. All genes were found to be in Hardy Weinberg equilibrium in the entire study population. This was also true when the subjects were divided into exposed and non-exposed groups, except for *SULT1A1*, which among exposed workers displayed more variant heterozygotes than expected.

In Table 2, all results that demonstrated statistical significance with respect to genotype-associated risk in either exposure-strata, or interaction between genotype and exposure status, are presented. For the majority of analyses, the unadjusted results were very similar to the adjusted. In the cases where the statistical significance differed, the unadjusted results are presented in the text below. For the other genotypes, no statistically significant associations were found.

**Table 2:** Symptoms of the eyes and airways comparing different genotypes among TDI-exposed and non-exposed workers.^a^

		**Geno-type**	**Symptoms**				
			**Yes (N)**	**No (N)**	**OR^b^**	**95% CI**	P^c*^
***EYES***							
***Exposed***	***NAT1***	IM+S	33	53	1.0	-	0.067
		R	23	17	**2.2**	**1.0–4.9**	
***Unexposed***	***NAT1***	IM+S	19	60	1.0	-	
		R	6	27	0.63	0.22–1.9	
***Exposed***	***SULT1A1 *R213H**	GG	27	14	1.0	**-**	0.19
		AG+AA	29	56	**0.22**	**0.096–0.52**	
***Unexposed***	***SULT1A1 *R213H**	GG	13	31	1.0		
		AG+AA	12	56	0.56	0.22–1.4	
***Exposed***	***TNF*-308**	GG	36	44	1.0	-	0.11
		GA+AA	17	19	1.0	0.47–2.4	
***Unexposed***	***TNF*-308**	GG	11	57	1.0	-	
		GA+AA	14	25	**2.6**	**1.0–7.0**	
***WHEEZE***							
***Exposed***	CYP1A1*2B	I/I	34	87	1.0	-	0.061
		I/V	5	1	**14**	**1.5–130**	
***Unexposed***	CYP1A1*2B	I/I	23	82	1.0	-	
		I/V	1	6	0.83	0.090–7.7	
***Exposed***	***SULT1A1 *R213H**	GG	17	25	1.0	-	0.58
		AG+AA	22	63	0.52	0.22–1.2	
***Unexposed***	***SULT1A1 *R213H**	GG	14	30	1.0	-	
		AG+AA	10	58	**0.38**	**0.15–0.99**	
***Exposed***	***CCL5 *-403**	GG	19	64	1.0	-	0.42
		AG+AA	20	21	**3.1**	**1.3–7.1**	
***Unexposed***	***CCL5 *-403**	GG	13	58	1.0	-	
		AG+AA	10	29	1.8	0.68–5.0	
***Exposed***	***TNF*-308**	GG	29	52	1.0	-	**0.043**
		GA+AA	7	30	**0.37**	**0.14–0.99**	
***Unexposed***	***TNF*-308**	GG	13	55	1.0	-	
		GA+AA	10	29	1.3	0.46–3.4	
***DRY COUGH***							
***Exposed***	HLADQB1*05	Absent	20	62	1.0	-	**0.047**
		Present	18	20	**2.7**	**1.2–6.3**	
***Unexposed***	HLADQB1*05	Absent	12	74	1.0	-	
		Present	1	23	0.27	0.031–2.2	
***Exposed***	***TNF*-308**	GG	30	50	1.0	-	**0.023**
		GA+AA	7	28	0.43	0.17–1.1	
***Unexposed***	***TNF*-308**	GG	6	62	1.0	-	
		GA+AA	7	32	2.2	0.66–7.3	
***COUGH WITH MUCUS***							
***Exposed***	***GSTM1***	Present	19	45	1.0	-	0.13
		Absent	24	39	1.4	0.66–3.0	
***Unexposed***	***GSTM1***	Present	8	55	1.0	-	
		Absent	18	31	**3.5**	**1.3–9.4**	
***Exposed***	***GSTM3***	AA	29	61	1.0	-	**0.028**
		AB+BB	14	23	1.2	0.52–2.7	
***Unexposed***	***GSTM3***	AA	23	53	1.0	-	
		AB+BB	3	33	**0.22**	**0.059–0.81**	
***Exposed***	GSTP1*105	I/I	26	41	1.0	-	**0.019**
		I/V+V/V	17	43	0.66	0.32–1.4	
***Unexposed***	GSTP1*105	I/I	8	48	1.0	-	
		I/V+V/V	18	38	**3.0**	**1.1–8.0**	
***Exposed***	***CCL5 *-403**	GG	22	59	1.0	-	0.079
		AG+AA	21	20	**2.9**	**1.3–6.5**	
***Unexposed***	***CCL5 *-403**	GG	17	54	1.0	-	
		AG+AA	8	31	0.86	0.32–2.3	
***NOSE BLEED***							
***Exposed***	***MPO***	GG	25	65	1.0	-	**0.018**
		AG+AA	3	30	**0.21**	**0.056–0.94**	
***Unexposed***	***MPO***	GG	3	70	1.0	-	
		AG+AA	4	35	2.9	0.58–14	

^a ^Adjusted effect estimates (for sex, age, atopy, and smoking) are presented as odds ratios (OR) and 95% confidence intervals. Statistically significant results are presented in bold.

^b^All results that reached statistical significance with respect to genotype-associated risk in either exposure-strata, or in the analysis of interaction between genotype and exposure status, are presented.

^c^Analysis of interaction between exposure and genotype was performed in a separate model using logistic regression with interaction term. P-values regarding interaction between exposure and genotype are indicated by P*.

**Figure 1:**
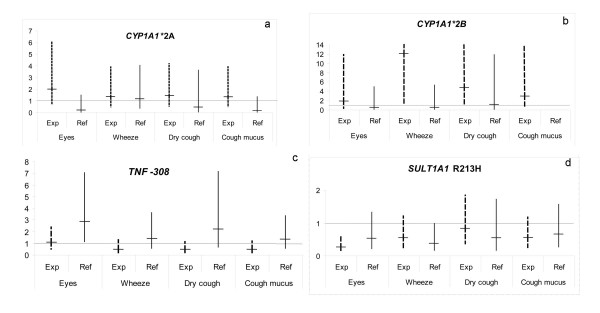
**a-d. Symptoms comparing different genotypes among TDI-exposed workers (exp) and non-exposed subjects (ref)**. Effect estimates are presented as crude odds ratios. Reference/variant genotypes are CYP1A1*2A A/B+C, CYP1A1*2B II/IV, *TNF*-308 GG/GA+AA and *SULT1A1 *R213H GG/GA+AA. A 95% confidence interval is indicated. Values above the Y-axis are indicated with Arabic numbers. Significant values are displayed in Table 2.

For three polymorphisms: CYP1A1*2A, CYP1A1*2B, and *TNF *-308 there was a pattern consistent with interaction between genotype and exposure status for the majority of symptoms investigated (Fig 1a–c). Among TDI-exposed subjects, the carriers of the CYP1A1*2A variant (crude: exposed workers OR = 1.9, 95% CI 0.62–5.8; non-exposed workers OR = 0.18, 95% 0.021–1.5; interaction p = 0.048 for symptoms of the eyes) and carriers of the CYP1A1*2B variant, had an increased risk for symptoms as compared to the non-exposed subjects. For *TNF*-308, the variant A allele was associated with a higher risk of symptoms among the non-exposed group, whereas the opposite was found to be true for exposed subjects. Among exposed subjects, the A variant allele was found to protect particularly against wheeze etc., but also against dry cough and cough with mucus (Fig 1c, wheeze etc.: crude OR = 0.54, 95% CI 0.22–1.3, interaction p = 0.130). For *SULT1A1 *R213H, there was no genotype exposure interaction, but the AG+AA carriers had reduced risk as compared to GG, both among exposed and non-exposed subjects (Fig 1d).

For other polymorphisms, associations were restricted to certain symptoms (Table 2). The rapid genotype of *NAT1 *was associated with a higher risk of symptoms of the eyes among exposed and interaction was indicated but not statistically significant. *CCL5 *AG+AA carriers had up to three times higher risk of wheeze etc. and cough with mucus among exposed workers. Carrying HLA-DQB1*05 was associated with an increased risk of dry cough among exposed subjects, and interaction was statistically significant. After subtyping for different *HLA*-*DQ05 *alleles, it was found that HLA-DQB1*0502 carriers, but not *0501, had an elevated risk of dry cough (*0502 OR = 4.9, 95% CI 1.1–21) among exposed workers. There were too few individuals with DQB1*0503 or *0504 for any association analysis.

Individuals with *GSTM3 *AB+BB were protected from cough with mucus among non-exposed subjects, with statistical significance for interaction. In the non-exposed group,*GSTM1 *null as well as GSTP1*105I/V+V/V individuals displayed significantly higher risk of cough with mucus. For *GSTP1*, evidence of genotype-exposure interaction was found as well. Among exposed subjects, *TNF*-863 CA+AA carriers had higher risk of cough with mucus than CC (unadjusted analysis OR = 2.5, 95% CI 1.1–5.6; adjusted analysis OR = 2.1 95% CI 0.91–5.0). Variant allele carriers of *MPO *were protected against nose bleed among exposed workers.

The gene-gene combinations analysis resulted in statistically significant associations between gene-gene combinations and symptoms. However, the results did not differ much from the results in the single gene analysis: e.g. for the combination *NAT1*+*SULT1A1 *and symptoms of the eyes, carriers of *NAT1 *IM+S and *SULT1A1 *AG+AA had an OR = 0.19 95% CI 0.066–0.53; for the combination of *TNF*-308 and *SULT1A1 *and wheeze etc., carriers of *TNF*-308 AG+AA and *SULT1A1 *AG+AA displayed an OR = 0.27 95% CI 0.081–0.91. There was no strong evidence for interaction apart from the combination of *GSTM1 *and *GSTP1 *for cough with mucus among non-exposed workers, where carriers of *GSTM1 *null and GSTP1*105 Val- displayed OR = 22, 95% CI 2.5–200, compared to *GSTM1 *present and GSTP1*105 Ile/Ile.

## Discussion

Gene-exposure interaction was observed for symptoms of the eyes and lower airways, whereas there were no strong indications of interaction for nose bleed. The *CYP1A1 *genotypes appeared to modify the effect of TDI. Similar pattern for the *Msp*I (*2A) variant as the Val (*2B) variant was found, which probably is due to linkage between these two polymorphisms [31]. However, the effect was strongest for *2B, possibly reflecting the higher biological relevance of this polymorphism. The difference between the wild type Ile and the Val variant appears to depend on the substrate: when the enzyme contained Val, ethoxyresorufin exhibited a mildly elevated activity, and estrogen exhibited a substantially elevated activity. No such effects were observed for benzo(a)pyrene [32,33]. There are no indications of effect of the *Msp*I variant alone on the CYP1A1 activity [34]. The role of CYP1A1 for TDI-related effects of the eyes and airways is at present largely unknown. In a murine model of TDI asthma, challenging with TDI resulted in an increased expression of CYP1A1 in the lung [35]. However, the role of CYP1A1 for TDI-asthma was unclear, since TDI seemed to partly inhibit the activity of CYP1A1.

Elevated levels of TNF are frequently observed in bronchoalveolar fluid of asthmatic subjects [36]. The -308G/A variant has been shown to be associated with elevated plasma TNF levels and with higher amounts of TNF upon stimulation [37]. The -863C/A variant is associated with reduced circulating levels of TNF [38]. In our study an increased risk of symptoms was indicated for the variant allele among the non-exposed group, which is in line with what has been reported in other studies for *TNF *-308 and non-occupational asthma [39]. However, we found that among the exposed subjects, the variant allele of *TNF *-308 was associated with reduced risk among the exposed whereas the variant of -863 was associated with increased risk. Thus, our results indicate that genotypes associated with low TNF circulating levels (-308 G and -863 A) may be linked to symptoms in the TDI-exposed. The impact of *TNF *polymorphism (-308) on TDI-asthma has been analyzed in a recent study by Beghé et al [40], but no associations were found in that study.

In *SULT1A1*, the variant allele of R213H (G/A), carries *in vitro *only 10–25% of the activity of the more common allele [41,42]. In our study, *SULT1A1 *showed significant effect on symptoms from the eyes and on wheezing, and a similar non-significant pattern was observed for all symptoms analyzed. With regards to eye symptoms, homozygotes for the more common allele G had up to three times higher risk than individuals heterozygous or homozygous for the low-activity A allele. However, the data did not suggest a gene-exposure interaction due to the fact that a protective effect was seen for exposed as well as for referents.

Higher *NAT1 *and *NAT2 *activity could hypothetically contribute to the formation of reactive damaging metabolites, by O-acetylation, in the eyes and airways. Hence, the rapid *NAT1 *and *NAT2 *genotypes could increase an individual's risk for TDI-associated symptoms. Gene-TDI interaction was suggested for *NAT1 *only for eye symptoms, where the rapid genotype showed approximately two times higher risk for symptoms among exposed workers. For NAT2 rapid acetylators, an increased risk was suggested for this symptom as well (OR = 2.17, 95%CI 0.99–4.79, p = 0.054). For the lower airways, an association between *NAT1 *slow genotype and increased risk for dry cough was found (data not shown), but it was only among non-exposed and there were no symptomatic exposed individuals with slow genotype, making it very difficult to evaluate the effect of this genotype. Thus, in contrast to Wikman and colleagues (14), we did not find any reliable associations with neither *NAT1 *nor *NAT2 *with the symptoms of the lower airways. The difference between the studies on isocyanate-exposed workers could be due to differences in selection of study subjects: in contrast to the study by Wikman et al., this is a cross-sectional study and the selection criterion was exposure and not disease. Moreover, we studied predominantly TDI-exposed individuals and did not include study subjects with a mixed diisocyanate exposure. Furthermore, in contrast to Wikman, we did not select asthma cases but rather analyzed symptoms which may be early indicators of asthma-related changes.

The -403G/A variant of *CCL5 *results in a new consensus element for the GATA transcription factor family [43], which suggests a role in the regulation of gene expression. In the present study, the *CCL5 *genotype appeared to have an effect on the risk for TDI-associated airway symptoms such as wheeze etc. and cough with mucus: the variant allele was associated with approximately three times higher risk among exposed, suggesting a CCL5-TDI interaction. An association for CCL5 in TDI-associated airway symptoms is also suggested from a recent study showing increased expression of CCL5 *in vitro *in bronchial epithelial cells when stimulated with TDI [44].

There was also evidence for association of *HLA*-DQB1*05, in particular the *0502 allele, and isocyanate-related symptoms of the lower airways. Compared to the *0501 allele, the *0502 has an exchange of a valine to a serine at amino acid position 57 in the beta chain of HLA class II molecules. Mapp et al. [15] have found an increased risk of asthma for *0503 carriers (in *0503, valine is substituted with aspartic acid at position 57), which we were unable to analyze, due to low number of study subjects carrying this allele. In contrast to Mapp, we did not find a protective effect for carriers of DQB1*0501 [15].

Glutathione S-transferases showed some effect on symptoms of the lower airways, in particular on cough with mucus. However, the effect was not observed among the exposed workers, but rather among the non-exposed subjects. One possibility is that these symptoms are caused by different mechanisms among exposed and non-exposed individuals, i.e. GSTs are not involved in TDI-related airway obstruction. Piirilä and co-workers [12], found an increased risk of diisocyanate-asthma among GSTM1*O subjects. We found only a non-significant higher risk of cough with mucus among the exposed subjects, and the strongest effect was found among non-exposed subjects. The differences in study design as discussed above for *NAT1 *and *NAT2 *could also explain differences in results for *GSTM1 *compared to Piirilä et al., since both Wikman's and Piirilä's reports are based on the same study population. Other studies have reported higher risk of non-occupational asthma for GSTM1*O subjects [45,46]. Piirilä also studied *GSTM3 *in relation to diisocyanate-asthma and found later reactions in the bronchial provocation test among GSTM3*AA individuals. We also found that *GSTM3 *was associated with airway symptom: there was a protective effect of GSTM3*B on cough with mucus. However, this was only evident among the non-exposed individuals. For *GSTP1*, the exposed I/V+V/V displayed a tendency towards a protective effect, which is in line with the study by Mapp et al. (13) on TDI-asthma and *GSTP1*. On the other hand, *GSTP1 *I/V+V/V subjects had an increased risk of cough with mucus among the non-exposed subjects.

Gene-gene interactions are of relevance to study, considering the complex metabolism of isocyanates as well as responses of the immune system. However, since the results differed for most polymorphisms for exposed and non-exposed, we had to analyse the gene-gene interactions in each group. The gene-gene combination analysis did not indicate any evidence for departure from an additive effect. The exception was the results from the combination of *GSTM1 *and *GSTP1 *for cough with mucus among non-exposed workers. Nevertheless, there may still be significant gene-gene interactions present for the symptoms analysed, however, for detection of such effects a larger study population had been required.

The low prevalence of certain risk genotypes are reflected in the large confidence interval for ORs obtained. Thus, the data should at this point be cautiously interpreted and new studies are needed for replication of the results. Another aspect to consider is the issue of multiple interferences. We examined fifteen polymorphic loci, and five symptoms for both exposed and non-exposed workers which might result in a large number of false positives. Nevertheless, no correction for multiple testing has been performed. Adjustments such as the Bonferroni correction are not applicable as they cannot take linkage between polymorphisms into account, which occurs among some genes investigated in our study. We do not stress individual significant findings, but rather believe that consistent association patterns between genotypes and symptoms indicate true findings.

By studying TDI-exposed as well as non-exposed individuals, specific gene-TDI interactions were indicated for symptoms of the eyes and lower airways. The finding that certain genotypes modified the effect of symptoms arising in different tissues suggests that these symptoms are associated with each other and have common mechanisms.

We did not expect to find individuals diagnosed with asthma among the exposed workers, since asthmatics should not be employed in isocyanate workplaces in Sweden today, and if workers acquire asthmatic symptoms after employment, they should be transferred to non-exposed jobs. Surprisingly, increased risks of severe symptoms of the airways, e.g., wheeze and/or chest tightness, which are associated with asthma (46), were recorded in the present study. Thus, bearing in mind that our study is a cross-sectional one, with potential for a healthy-worker-selection, our findings may be an underestimation of the true risk. Furthermore, gene-exposure interactions appear to be present in early signs of health effects.

## Competing interests

The authors declare that they have no competing interests.

## Authors' contributions

KB carried out part of the molecular genetic analyses, performed part of the statistical analyses and drafted the manuscript. HT carried out the exposure assessments and participated in the design of the study. AA performed part of the statistical analyses. MW and AR carried out part of the molecular analyses and the design of the study. ML conceived of the study, participated in its design and coordination, and performed the health investigations. All authors read and approved the final manuscript.
